# “One program that could improve health in this neighbourhood is ____?” using concept mapping to engage communities as part of a health and human services needs assessment

**DOI:** 10.1186/s12913-018-2936-x

**Published:** 2018-03-01

**Authors:** Alisa J. Velonis, Agnes Molnar, Nakia Lee-Foon, Ashnoor Rahim, Mary Boushel, Patricia O’Campo

**Affiliations:** 1 Centre for Urban Health Solutions, St. Michael’s Hospital, 30 Bond Street, Toronto, ON M5B 1W8 Canada; 2 University of Illinois at Chicago School of Public Health, Division of Community Health Sciences, 1603 W Taylor St, Chicago, IL 60612 USA; 3 Toronto Central Local Health Integration Network, Toronto, ON Canada; 4 University of Toronto, Dalla Lana School of Public Health, Toronto, ON Canada; 5 WoodGreen Community Services, 815 Danforth Ave Suite 100, Toronto, ON M4J 1L2 Canada; 6 Health Quality Ontario, 130 Bloor St W, Toronto, ON M5S 1N5 Canada

**Keywords:** Community engagement, Community health services assessment, Concept mapping, Health services planning

## Abstract

**Background:**

This paper presents the findings of a rapid needs assessment conducted at the request of the local health authority responsible for health care services, the Toronto Central Local Health Integration Network (Ontario, Canada), to inform health and social service planning.

**Methods:**

We utilized concept mapping methodology to facilitate engagement with diverse stakeholders–more than 300 community members and service providers–with a focus on hard to reach populations. Key informant interviews with service providers were used to augment findings.

**Results:**

Participants identified 48 unique services or service approaches they believed would improve the health of residents in the area, including those addressing health care, mental health and addictions, youth, families, people experiencing homelessness, seniors, general social services, and services targeting specific populations. While service providers consistently identified a critical need for mental health and addiction services, community members placed greater importance on the social determinants of health including access to housing, job placement supports and training and service accessibility. Both groups agreed that services and programs for seniors and people experiencing homelessness would be highly important.

**Conclusion:**

Our study provides a unique example of using concept mapping as a tool to aid a rapid service gap analysis and community engagement in a metropolitan area. The findings also reinforce the importance of working cross-sectorally, using a Health in All Policies approach when planning services for underserved populations.

## Background

Across the health services planning literature, the process of engaging community residents and specific targeted populations has been touted as a useful – if not essential – part of effective planning and evaluation [1–4]. Across the globe, public health departments, hospital systems, and social service planners are increasingly using focus groups, community forums, door-to-door surveys, and similar activities to gather the input of non-professionals and residents into long-term and strategic planning initiatives, to better involve them in decision-making [5–7]. These forms of gathering input recognize the importance of engaging in health planning with – rather than for – communities [5–7]. As the vantage point of the populations directly affected by health problems (and those for whom intervention and prevention programs are directed) is included and valued, community-engaged planning processes are particularly effective at identifying and addressing the social inequities that often result in health disparities and illness [8, 9]. Concept mapping is one such method to capture populations’ feedback.

Concept mapping (CM) is a participatory, mixed-methods approach that can be used to generate or conceptualize ideas, illustrate relationships between ideas, and rate ideas using a variety of criteria [10–12]. Data are collected and synthesized in three primary phases: brainstorming, sorting and rating, and mapping. Throughout these phases, an iterative process exists between the researcher(s) and participants as data moves back and forth between the two groups, with the final validation step resting with participants. Two of the strengths of this approach as a participatory research and planning tool include: (a) its ability to gather and synthesize input from multiple sources with different expertise and backgrounds, and (b) the limited nature researchers have over the data, analysis, and final results [12]. Concept mapping has been used in a variety of capacities within the health services planning and evaluation field, from identifying characteristics or behaviors that signify health or well-being [13], elements that should be included in new programs approaches to service delivery [14–16], and to the development of a national public health strategic plan for addressing cognitive health [13–17]. Although the literature contains many examples of using CM to engage predefined groups of people (such as parents, teachers, students, and staff within a school or diabetic patients affiliated with a particular health system) [18–20], there are few examples of its use as a tool in regional or neighbourhood community-engaged needs assessments, and almost none in the peer-reviewed literature. Minh et al. (2015) used CM to conduct a needs assessment for youth in Toronto [21], and in the U.S., the team found one hospital system that incorporated CM into its community health assessment process, asking a community advisory council to identify the biggest health care problems and then engaging community residents in a subsequent rating and prioritization process [22].

This paper describes how concept mapping processes were integrated into a health and social services needs assessment to conduct a rapid yet participatory assessment of the needs within a geographically broad and demographically diverse area of Toronto, Ontario.

### The project

The use of concept mapping in health services planning is not new in Ontario, Canada. Since 2006, the oversight of health care in the province has been regionalized into 14 Local Health Integration Networks (LHINs), which are responsible for health services planning and delivery. Community engagement (CE) is a required part of this planning, and between 2008 and 2009, researchers and LHIN employees used concept mapping to explore how each of the LHINs conceptualized CE and developed a framework for future CE evaluation [23].

As a demonstration of this commitment to community engagement, in 2014, the Toronto Central LHIN (TC-LHIN) employed a local research center to conduct a needs assessment for a geographically-defined community near Toronto’s downtown core. According to the 2011 census, which collects data via the internet, phone, mail and one-on-one interviews [24], approximately 124,960 residents lived within the area, representing a 9.7% increase in population growth between 2006 and 2011 [25, 26]. The area included a high proportion of adults 20–44 years (47.4%), a low proportion of children 0–14 years (9.8%) and one of the highest proportions of seniors living alone compared to other parts of central Toronto. Almost 20% of residents spoke a primary language other than English, most commonly Mandarin, Cantonese, Chinese (nonspecific), Bengali, Tagolog, Spanish, and Tamil, and over 9% of the population immigrated to Canada in the previous five years [26]. This area is characterized by wide economic inequities, with average family incomes ranging from $39,521 to $365,211. The scope of this assessment focused on individuals and families in the lower range of the spectrum, as 26% of individuals in private households lived below the poverty line [26]. Data obtained by the TC-LHIN (Mid Toronto East Health Link, unpublished report, TC-LHIN, 2013) show that residents in the area faced a diverse array of health issues, including high rates of diabetes, asthma, chronic obstructive pulmonary disease, high blood pressure and low rates of mammograms, Pap smears, and other common health screenings. Leading causes of premature mortality, particularly in the low-income areas, included heart disease, lung cancer, HIV/AIDS, and intentional self-harm (Mid Toronto East Health Link, unpublished report, TC-LHIN, 2013). High utilization rates of emergency and mental and addiction health services were characteristic of this area (Mid Toronto East Health Link, unpublished report, TC-LHIN, 2013).

In response to the opening of a new medical clinic in the area, health planners from the funding agency wanted to know which social and mental health services area residents and service providers believed should also be available at the clinic. Upon the request of funder, the research team (AV, AM, NLF, PO) sought the opinions of key informants and area residents from several “priority populations.” These populations included youth, seniors, individuals of Indigenous decent, immigrants, self-identified LGBT, Mandarin and Bengali speakers, and individuals experiencing mental health or addictions issues. The research team collected opinions through a rapid assessment using concept mapping techniques with area residents and one-on-one interviews with key informants. Two main questions drove the research:

i) What are the needs and gaps in health and social services as perceived by residents and service providers?
ii) Do these perceived needs and gaps differ by subgroup (e.g., service providers, residents, or identified priority populations)?

## Methods

### Data collection

#### Concept mapping

Concept mapping (CM) is a structured conceptualization process consisting of three phases: i) brainstorming ideas in response to a focal question, ii) rating the importance of the different brainstorming ideas, and iii) describing how these ideas are interconnected. Concept mapping is a semi-quantitative, participatory research method in which every participant’s voice counts in determining how a group views a particular topic or aspect of a topic [10, 27, 28]. Participants serve as experts because of their membership in specific groups of interest; in this case, this included residents from the neighbourhoods, members of the identified priority populations, health care providers, and representatives of social service organizations. The priority for this project, because of the very short turnaround time, was on the first two activities, brainstorming ideas and rating items and is the focus of this paper.

A community engagement plan was developed to ensure that feedback from community members, health and service providers and those using services in the region was collected. Six *community animators,* individuals knowledgeable of the neighourhoods in question, were hired to assist the research team with data collection. The animators used their familiarity with the area to identify locations (e.g., community organizations, clinics, or businesses) where members of the priority population congregate in order to directly recruit participants or foster connections with key representatives. The animators visited these neighbourhoods each day of the week, including weekends. Community animators:

- Distributed flyers and information sheets to potential participants;
- Conducted encounter interviews on the spot, gathering examples of programs and services during the brainstorming phase and later administering the rating sheets;
- Recruited participants for group brainstorming sessions;
- Recruited participants to complete the on-line surveys;
- Visited community organizations to engage staff and participants and hold on-site brainstorming sessions.

Data collection and analysis occurred in two phases over approximately 5 weeks in 2015. In phase one, participants took part in one-on one brainstorming sessions with community animators, facilitated group sessions, or electronically using email or through Concept Systems Global Max© software. Participants were told about the plans to provide new services and were asked to respond to the following question: *One program or service that would improve the health of the residents living in [this area of the city] is___________________________.* Participants were encouraged to provide as many responses to this question as they wished. The research team then systematically condensed over 250 responses to 48 by eliminating duplicate responses and combining similar ideas. In phase two, participants were asked to rate each item on a 6-point Likert scale based on the degree to which the item would improve individuals’ health and how frequently the programs or services would be used (see Table 1). Participants who attended the facilitated group sessions were reimbursed for travel in the form of transit tokens, and light snacks and beverages were provided. The sessions and online surveys were conducted in English, and some group sessions were held in Mandarin, one of the most non-English languages spoken in the area, using a translator.

**Table 1:** Rating statements and response categories

*Outcome*	*Rating statement*	*Response category*
Health improvement	This program or service would greatly improve the health of residents in the community	1 = Completely Disagree, 2 = Strongly Disagree, 3 = Somewhat Disagree, 4 = Somewhat Agree, 5 = Strongly Agree, 6 = Completely Agree
Frequent use	This program or service would be used very often by residents	1 = Completely Disagree, 2 = Strongly Disagree, 3 = Somewhat Disagree, 4 = Somewhat Agree, 5 = Strongly Agree, 6 = Completely Agree

Regardless of specific activity or the setting, all participants who took part in rating were asked to complete a short demographic survey. The survey captured information on participants’ age, gender, education, whether they were born in Canada or were immigrants, where they currently live, and how they were connected to the community (e.g. resident, service provider, employed in the area, etc.). A map of the catchment area was available to clarify the geographic boundaries. Participants did not include any identifying information on the questionnaire.

#### Key informant interviews

As a complementary process to the concept mapping activities, individual interviews were conducted in person or by phone with key representatives of health and social service organizations. The individuals interviewed held leadership roles (e.g. director, CEO, lead physician, manager) at these organizations and help determine the allocation of resources as well as the capacity to organize/deliver services. The purpose of conducting these interviews was to supplement the concept mapping information with data that illustrated the perceptions and priorities of decision-makers within the organizations that currently served the area. Interviews were approximately 20 min in length and consisted of open-ended questions and probes for further clarification, when necessary.

#### Ethics

All participants provided verbal consent to participate. This was in accordance with the Research Ethics Board of St. Michael’s Hospital (protocol #14–052), based on a desire to protect the anonymity of the concept mapping participants and because the risks involved with participation were minimal.

### Analysis

#### Concept mapping

Concept mapping data were entered into the Concept Systems Global Max© software and subject to hierarchal cluster analyses and multidimensional scaling. “Go-zone” figures [10] were used to visually display how service providers and community members perceived the importance of services by both its likelihood to improve health and to be frequently used. “Pattern Match” figures [10] allowed us to display the correlation between the ratings of two participant groups. A correlation coefficient (r) provided a statistical measure of the overall relationship between the ratings of services or participant groups according to the two rating questions.

#### Key informant interviews

Key informant interviews were audio recorded and transcribed verbatim, and all identifying information was removed. Using content analysis approach [29], and hand coding, investigators identified main themes related to needs and gaps in the service system and priority populations.

## Results

### Concept mapping

#### Participants

Over 150 participants took part in phase one and nearly 200 participants took part in phase two of concept mapping**.** As the brainstorming phase of concept mapping generates collective rather than individual data, individual-level collection of demographic data was limited to those who took part in the rating exercises, and of these, approximately 80% completed a demographic survey. Of the participants who completed rating forms for at least one of the three scales and provided demographic data (*n* = 144), almost 62% reported living or working in the area, and 61% said they received health or social services within the community. Sixteen percent of participants indicated they were health or social services providers in the area. Roughly 20% of participants were youth under 18 years of age, over half identified as female, and almost 40% were immigrants to Canada. While 20% of individuals reported earning a Bachelor’s degree or higher, almost another 30% of participants had not completed high school. Please see Table 2 for an overview of participant demographics.

**Table 2:** Demographic Data for Rating Participants Who Completed Demographic Questions

Participant Demographics (*N* = 151)	**%** ^**a**^
Age	
16–17 years	18.83
25–54 years	54.54
55–64 years	25.97
Gender	
Male	44.16
Female	53.9
Transgender	1.3
Canadian born	61.04
Education	
Some high school	26.62
High school graduate or equivalent	16.23
Some college/university	24.03
Bachelor’s degree	20.13
Graduate degree	9.74

^a^Due to missing data, percentages do not add up to 100

During the brainstorming phase, over 250 responses were recorded, which the research team reduced to 48 unique items by eliminating duplicates and combining similar ideas into a single item. Our final list included specific services or programs, (e.g., walk in dental clinic), services for specific populations (e.g., in-home care for seniors), and desired characteristics of programs (e.g., culturally appropriate health care services). The items were organized into nine clusters representing related concepts: health care, mental health and addictions, youth, family, social services, community and recreation, homeless, seniors, and other populations. The left-side of Table 3 shows a complete list of the final 48 services, organized by cluster.

**Table 3:** Forty-Eight Services & Average Ratings within Their Clusters^a^

*Cluster and Item Names*	*Health improvement rating*		*Frequency of use rating*	
	*Service providers*	*Community members*	*Service providers*	*Community members*
*Health Care*				
3. Pediatricians.	4.08	4.44	4.12	4.35
6. An emergency or urgent care clinic (staffed with physicians and open in the evening hours).	4.76	4.99	4.82	4.87
9. A sexual health clinic (offers cervical cancer tests, prenatal care, HIV prevention services).	4.72	4.69	4.41	4.52
19. A service that helps patients coordinate health care services and referrals.	4.40	4.59	4.53	4.93 (3)
21. Health services that are culturally appropriate and available in many languages.	4.44	4.74	4.76	4.60
23. On-site outpatient specialist health services (e.g asthma specialists and pulmonologist, stroke and diabetes care).	4.52	4.96	4.59	4.78
27. A program that teaches people with disabilities to live independently.	4.36	4.83	4.06	4.67
31. A walk-in medical clinic that is open 24/7.	5.00	5.03 (5)	4.88	4.77
38. A walk-in dental clinic (including the ability to see patients without dental coverage).	5.08	4.91	5.18 (5)^c^	4.83
44. Nutrition counseling (education sessions and workshops).	4.44	4.41	4.12	4.43
45. Alternative/naturopathic services and training.	4.08	4.39	4.06	4.47
*Mental Health and Addictions*				
4. Mental health services that are accessible after hours, no waiting list, geared to income.	5.48 (1)^b^	4.9	5.53 (2)	4.73
8. Drug and Alcohol Counseling that is accessible after-hours, with no waiting list, and geared to income.	5.32 (2)	4.72	5.12	4.55
18. Harm reduction program for substance abuse users (clean needles, safe injection site, etc.).	4.56	4.59	4.71	4.75
29. Drop-in counseling (including treatment for specific mental health problems such as chronic depression, grief, post-traumatic stress disorder).	5.16 (5)	4.87	5.35 (3)	4.70
41. A mental health crisis centre that is open after hours.	4.24	4.73	5.59 (1)	4.72
*Youth*				
2. After-school and summer programs for children and youth.	4.76	4.87	4.76	4.57
11. Mental health and substance abuse program for street-involved youth.	5.2 (4)	4.78	4.94	4.83
16. Counseling services for youth (should address issues like depressing, bullying, etc.).	4.84	4.77	4.71	4.75
22. Educational support programs for youth (tutoring, literacy intervention, college/university application assistance).	4.40	4.81	4.47	4.78
35. A “recreational centre” for youth (a safe place with no weapons, open on weekends, where youth meetings can be held).	4.20	4.82	4.24	4.70
36. A walk-in health clinic for youth (open after hours, including sexual health services).	4.92	4.83	5.00	4.63
39. Sports and recreational program for youth (organized by age).	3.76	4.51	3.88	4.6
40. Comprehensive sexuality education programs for youth.	4.36	4.46	4.18	4.48
*Family*				
14. Counseling and support services for teen moms.	4.72	4.82	4.35	4.7
34. A child care program (affordable, full day).	4.48	4.71	4.88	4.87
42. Parenting support and education (gymboree, support groups, cooking classes).	4.12	4.49	4.29	4.45
*Social*				
7. A food bank (one that does not require identification.	4.24	4.88	4.35	4.88 (5)
13. Help finding affordable housing (e.g case managers).	4.92	5.11 (1)	4.94	4.98 (1)
20. Adult educational services (e.g high school upgrading, literacy classes, life-skills classes, ESL classes).	4.20	4.79	4.35	4.82
24. Job training (e.g youth programs or program that helps people find both temporary and permanent jobs, internships, and apprenticeships).	4.28	4.90	4.35	4.92 (4)
*Community Centre and Recreation*				
5. Free, low-cost, accessible space that community members and service organizations can reserve for community gatherings.	5.08	4.82	4.41	4.63
10. A place where people can come to socialize (e.g play games like foosball and table tennis and dance).	4.20	4.82	4.12	4.58
28. A “hub” where social service organizations can provide services on a rotating basis (e.g on Mondays Agency A is there, on Tuesday’s, Agency B is there).	4.36	4.57	4.41	4.67
30. A quiet space with computers and WiFi that can be used by the community.	3.76	4.54	4.06	4.55
37. Fitness classes for parents and children (for example, free yoga classes).	3.92	4.56	4.06	4.50
47. A fitness centre (for example, a place with exercise machines).	3.76	4.50	3.94	4.42
*Individuals Experiencing Homelessness*				
1. Mental health services for homeless individuals.	5.48 (1)^b^	5.07 (3)	5.24 (4)	4.77
17. Health services for the homeless (e.g treating cold and foot problems).	4.84	4.92	5	4.95 (2)
26. A place open 24 h/day where those who need to can come to stay warm, take showers, use clean washrooms, do laundry and sleep.	4.72	4.89	5.18 (5)^‡^	4.80
*Seniors*				
12. Outreach to help isolated seniors access health care and social services.	5.28 (3)	5.06 (4)	4.94	4.83
25. In-home care and assistance for seniors.	4.52	4.87	5.06	4.8
32. Assistance for seniors who need help getting to and from appointments.	4.64	4.91	4.71	4.57
43. A program that provides classes and social events for seniors (e.g. fitness classes for seniors, English-as-a-second-language classes, teas, etc.).	4.24	4.76	4.12	4.77
*Specific populations*				
15. LGBTQ services/programs, drop-in programs, weekly programs.	4.32	4.47	4.24	4.43
33. Services for women or families experiencing domestic violence.	4.64	5.08 (2)	4.65	4.68
46. A safe place specifically for women (for example, where women can drop-in during the day and where women’s groups can meet).	4.56	4.87	4.41	4.75
48. Health services for Indigenous people.	4.08	4.51	4.24	4.72

^a^The rankings of the highest 5 items in each column are shown in parentheses

^b^These items are tied for importance to improving health as rated by community members

^c^These items are tied for frequency as rated by service providers

Table 3 illustrates how both service providers and community members rated the 48 items in terms of their importance to health and the frequency they would likely be used (ranks of top rated items are shown in parentheses). A key finding is that community residents and service providers held different perspectives on what was most critically needed. Service providers ranked four of the five mental health services as the highest in importance to improve health. Conversely, community members’ top ratings only included one mental health item – mental health services for those experiencing homelessness – as a service that would improve health. Further, community members clearly identified non-clinical social services as those that would be used frequently and of greater benefit to health. These services included assistance finding affordable housing, job training, food banks, and services for domestic violence victims and seniors, as well as efforts to increase the accessibility of health care services to specific populations.

**Fig. 1:**
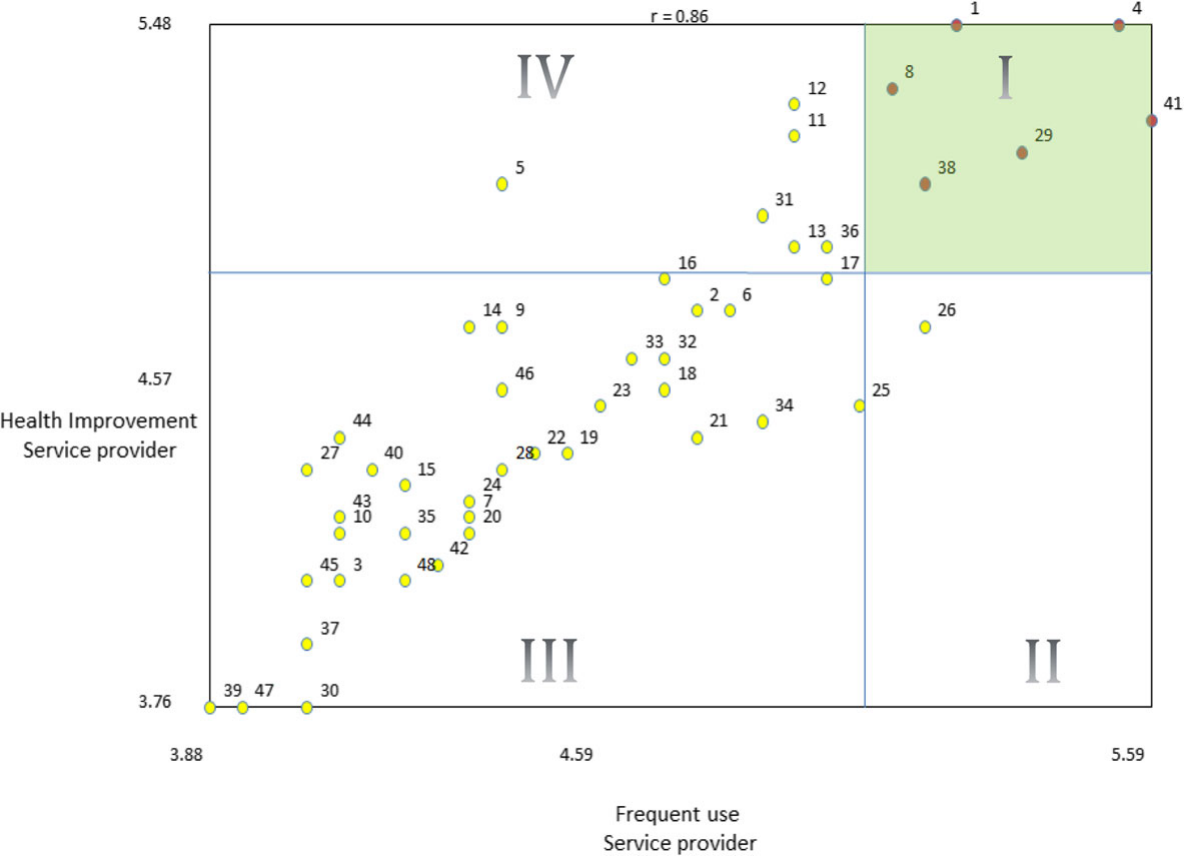
Go-Zone illustration of service provider rating by frequency of use and likelihood to improve health. (a) Each numbered point refers to a specific statement (see Table 3 for complete list of statements and corresponding item numbers). For example, the point labeled 30 represents the statement “A quiet space with computers and WiFi that can be used by the community.” (b) Quadrant I (green) contains items which are both most likely to improve health and most likely to be used by members of the community. Quadrant II contains items which are less likely to improve health, although are likely to be used frequently. Quadrant III contains items ranked lowest in terms of frequency of use and effectiveness on health. Quadrant IV contains items that may contribute to health improvement, but would not be used very often

**Fig. 2:**
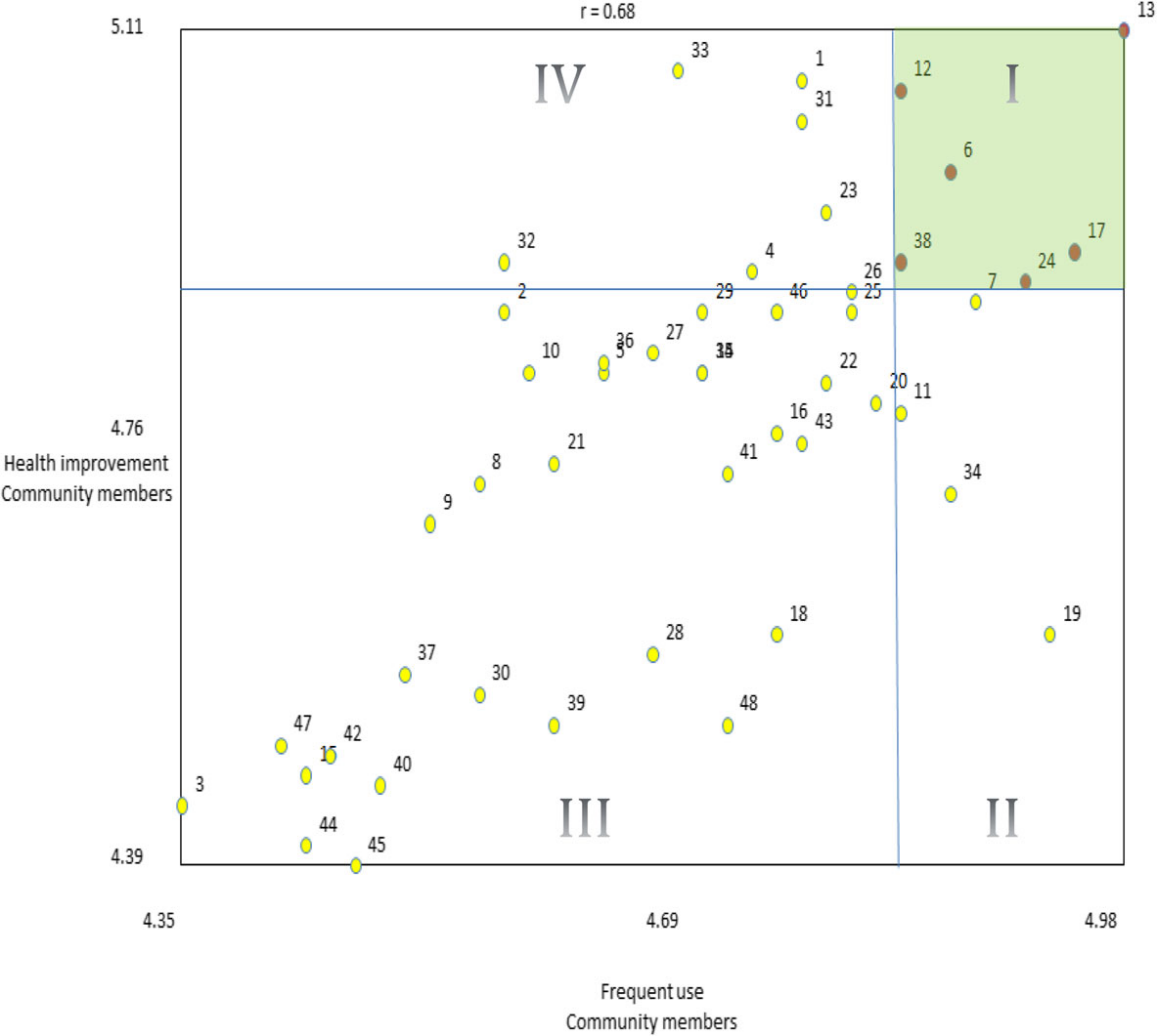
Go-Zone illustration of community members rating by frequency of use and likelihood to improve health. (a) Each numbered point refers to a specific statement (see Table 3 for complete list of statements and corresponding item numbers). For example, the point labeled 30 represents the statement “A quiet space with computers and WiFi that can be used by the community.” (b) Quadrant I (green) contains items which are both most likely to improve health and most likely to be used by members of the community. Quadrant II contains items which are less likely to improve health, although are likely to be used frequently. Quadrant III contains items ranked lowest in terms of frequency of use and effectiveness on health. Quadrant IV contains items that may contribute to health improvement, but would not be used very often

Using go-zones**,** which compare ratings using a bivariate graph divided into quadrants determined by dividing above or below each variable’s mean score [10], the team examined the relationship between the likelihood a service would improve health and the likelihood that the service would be used, looking separately at service providers’ and community members’ responses (Figs. 1 and 2). In both figures, the green-shaded quadrant (I) represents the items which are both most likely to improve health and most likely to be used by members of the community. This area represents the “Go-Zone,” where effort may be more likely to pay off (e.g., not only will helping community members find affordable housing (item 13) positively contribute to their health, but it would be used frequently). Quadrant II contains those items which are less likely to improve health, although they would be likely to be used frequently. Quadrant III items were ranked lowest in terms of frequency of use and effectiveness on health, and Quadrant IV illustrates those items that may contribute to health improvement, but would not be used very often. Again, service providers placed particular importance on mental health services, whereas community members focused on social determinants of health, including assistance finding affordable housing, job placement and training, food banks, services for women experiencing domestic violence and services for seniors (See Table 4 for list of top ranked services or service approaches by participant type based on the average rating scores across both questions). Service providers’ perspectives correlated more than the community members.

**Table 4:** Top rated services or service approaches by participant type ^a^

*Service Providers*			*Community Members*		
Average Rank Order	*Q1* ^***c***^ *rank*	Q2^d^ *rank*	Average Rank Order	*Q1* ^***c***^ *rank*	Q2^d^ *rank*
4. Mental health services that are accessible after hours, no waiting list, geared to income.^b^	1	2	13. Help finding affordable housing (e.g., case managers).	1	1
1. Mental health services for people experiencing homelessness.^b^	1	4	12. Outreach to help isolated seniors access health care and social services.	4	8
29. Drop-in counselling (including treatment for specific mental health problems such as chronic depression, grief, post-traumatic stress disorder).	5	3	17. Health services for people experiencing homelessness (e.g., treating cold and foot problems)	8	2
8. Drug and Alcohol counselling that is accessible after hours, with no waiting list, and geared to income.	2	6	6. An emergency or urgent care clinic (staffed with physicians and open in the evening hours).	6	6
38. A walk-in dental clinic (including the ability to see patients without dental coverage).	7	5	1. Mental health services for people experiencing homelessness.	3	16
12. Outreach to help isolated seniors access health care and social services	3	11	24. Job training (e.g., youth programs or program that helps people find both temporary and permanent jobs, internships, and apprenticeships).	12	4

^a^The order of items in the chart is based on the rank order of the average of both rating questions

^b^Items 1 and 4 were tied in importance to health improvement by service providers

^**c**^Q1 shows the ranking for “health improvement”

^d^Q2 shows the ranking for “frequent usage”

Finally, the team examined differences between service providers’ and community members’ categorical priorities using pattern matching. While both groups ranked *individuals experiencing homelessness* and *senior services* as important, service providers perceived *mental health and addiction* as the most important cluster of services to improve health, which greatly differed from community members who ranked services for *people facing homelessness* as the most important, followed by *social services*. Both service providers and community members agreed that *health care* was one of the least important clusters.

**Fig. 3:**
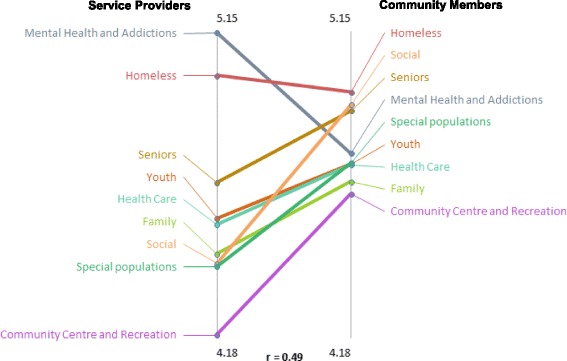
Pattern match illustrating health improvement cluster ratings for service providers compared to community members

Community members tended to rank items as close in importance to each other (illustrated by the tight clustering of services on the right side of Fig. 3). Conversely, service providers perceived some services were more important than others. Findings were similar when the team examined the rating of service clusters that would be *frequently used* by the residents of the community (not shown).

As different populations have unique needs, the team also investigated whether perceptions about the frequency with which certain services would be used differed by participant demographics. For example, while both Canadian-born and immigrant participants rated health and mental health services as having equal levels of importance, Canadian-born participants believed that services for people experiencing homelessness would be used with greater frequency than non-Canadian born participants, whereas the latter group prioritized services for seniors. Likewise, people with less than a high school education ranked the social cluster the highest or most likely to be used with greater frequency, those with at least a high school education believed that mental health and addiction and senior services would be frequently accessed.

#### Key informant interviews

Eighteen key informants were interviewed, representing the leadership of 7 types of organizations: community health centres; general hospitals; primary care providers; community health, mental health and social service providers; and community care access centres. Key themes that emerged included the need for improved access to services for individuals experiencing homelessness, those living with mental health problems, and those facing multiple health problems and challenging social conditions. Services for immigrants were also cited as requiring attention, and language barriers were identified as central. Finally, respondents identified a need for more coordination and integration of health and social services. These interviews confirmed many CM findings such as the needs of priority populations that were captured in the brainstorming phase of concept mapping. Several strategies to improve access and utilization of services were also discussed, including making services easier to apply for and increasing availability outside of traditional office hours.

When key informants were asked about service gaps, many believed the area already had a wealth of programs and services; however, the extent and nature of these services needed to be clarified for residents and other service providers. As one participant noted:

“*I just think the issue is not that the services aren’t there, it’s, for whatever reason, the coordination just hasn’t been there between all the organizations in [the neighborhood]. I think a lot of it is, we have the money for the priority health care, we don’t have the extra resources for self-promotion and community engagement.”*

Concerning the issue of health care, many informants noted that access to stable, continuous primary care was the most important area of focus to improve health. As one participant said,

*“I think the first requirement is access to stable, continuous primary care. I think we need to look at it in flexible ways, in evenings and weekends, because I think that is the way to avoid, prevent the use of the emergency department.”*

This was consistent with the view that more outreach into the community via methods such as mobile staffing and satellite offices would also increase access by community members. One key informant noted these methods would be beneficial for hard to reach patients and supportive to family physicians.

While community mental health programs are well provided, many believe that they are not well connected with psychiatry and there is a serious need for community psychiatric support. Many key informants identified a gap in the provision of urgent psychiatric care to support primary care practitioners and to meet the needs of the population in general, as well as for specialized groups such as children, seniors and individuals with a mental health diagnosis. There was also a strong need for psychiatric assessment and greater support for medication adherence.

## Discussion

This paper presents the findings of a rapid needs assessment which integrated several concept mapping methods into a community engagement process. By doing so, the team was able to include the input from a wide range of populations, including many who are often left out of health services planning, such as individuals experiencing homeless, immigrants, youth, seniors, and those with mental health and addiction issues [30–32]. This process also allowed our team to identify discrepancies between the opinions of professional service providers and those of community members about health and social service priorities in the area.

Considering that neither group viewed any service or category to be of low importance (indicating a need for a wide range of services), this distinction in perspective is critical from the standpoint of resource allocation and services planning. Whereas service providers consistently identified mental health and addiction programs as most critical to health and as those that would be frequently used, community members spoke strongly about gaps in services that address the social determinants of health. It could be argued that service providers’ identification of these programs reflects greater awareness among providers about mental health problems and existing strategies and programs; and that providers working in the field of mental health and addictions or whose clients struggle with psychological issues and substance abuse are likely to focus on the numerous gaps in these programs.

Conversely, by rating *social* rather than *clinical* services so highly, the individuals who are most likely to *use* these services may be acknowledging a very practical reality, that before specific services focused on substance abuse, mental health, or physical well-being can be effectively addressed, people need stable housing, food, and adequate safety as well as services that can be accessed when and where they people need them. The key informant interviews reinforce this last point as well. Although focused on service provision, many informants echoed the sentiment that it would be most beneficial to improve the coordination and integration of pre-existing services rather than create new services.

It is clear that none of the 48 services were considered unimportant to the community, although some were ranked higher than others. Moreover, the findings reinforce several key areas of importance. First*, if asked*, *community residents think about health broadly and understand that social determinants are as important as clinical services*. While this may seem simplistic, even when community health and needs assessments use community-engaged methods, many traditional approaches only ask residents to prioritize health problems rather than identify solutions [33, 34]. Yet when asked, residents clearly recognize that social and structural inequities, including unemployment, housing and food instability, and violence are at the root of community health [35]. This clearly suggests service providers, policy makers, and health officials need to work cross-sectorally, and promote a ‘Health in All Policies’ approach to find ways to meet core needs, such as housing and safety to impact the overall health status and health system costs for the population.

Second*, better coordination and communication between clinical and social services are essential* [36]. In terms of next steps, the compatibility of the services placed in one facility should be considered, along with the physical set-up of the space and with the potential opportunities for better coordination and collaboration between programs and providers in a shared space [37, 38]. In case of planning multiple services, either by proposing shared space for multiple services or making services available on a rotating basis, providing client case management or services coordination could be especially beneficial to clients with multiple service needs. The project’s funder intends to use the findings of the assessment plan to determine which social or mental health service should be included in the clinic space.

Finally, *this article advocates for the use of concept mapping as an innovative participatory strategy for community health assessment and improvement planning*. In the U.S., community health needs assessments and improvement plans are required of health care organizations and public health agencies to maintain tax-exempt status and accreditation [1, 39, 40]. Widely-used assessment frameworks such as The Mobilizing for Action through Planning and Partnership process [41] place community engagement squarely at the core of effective assessment, yet these assessments, when done using common approaches to focus groups and community surveys, run the risk of only involving the partners who are traditionally involved in service delivery, without truly engaging a broad spectrum of community residents [42, 43]. The work presented here, as well as that of Burke and colleagues [22] illustrates how concept mapping methods can be used to engage a large and diverse cross-section of communities in the process of assessing and prioritizing issues related to health. In particular, this method was a rapid and efficient means of incorporating the perspectives and preferences of many of the priority populations for the Local Health Integration Network such as those who are not housed or those who do not speak any of the official languages. Time and expense are often cited as a reason to not engage broadly with priority populations, and true community based participatory research methods are often labor, time, and resource intensive to do well [42, 44]. Concept mapping activities could be a means to overcome these barriers.

### Limitations and strengths

Limitations of the project should be briefly noted. *First, the study was launched with a condensed time frame* in which to collect these data, which limited the approaches to recruitment (e.g., scheduled group events with sufficient time for RSVP and reminder calls); possibilities to involve more participants from specific priority groups (e.g., from non-English speaking populations); and to explore service gaps across these groups. The short timeframe and logistics of engaging participants in community settings also limited our methodology, and the team did not complete the mapping phase usually done during concept mapping. This was unfortunate, as it did not allow the research team to hear how community members themselves conceptualized the findings and to learn if they would have interpreted the differences between providers and residents in the same way. *Second, the project focused on perceived gaps in services.* The method the team chose prevented us from documenting whether gaps that surfaced were a result of a lack of availability or due to barriers caused by a lack of coordination between different providers/services/programs.

Our study also had several strengths. Despite the short time frame, researchers managed to engage widely throughout the community, reaching out to several hundred residents and service providers within the area, as a result of a targeted recruitment process. Usually when communities are consulted, residents are asked to come to a meeting to provide input, severely limiting the population providing feedback [42, 44]. In our case, community animators went to the community, to places where residents lived, worked, shopped, and received services. This enabled us to involve multiple segments of individuals in the area including those who may be unlikely to participate in after-hours focus groups or to complete and return a survey. In addition, planners from the funding agency helped to reach out to their service providers in the area and engage them to participate in the study. This way, our sample represents a population that may not be generalizable to the entire region, but consists of those who use and/or provide services and was ideal for this gap analysis.

## Conclusions

These findings demonstrate the importance of planners going into communities, where people live, work, and play, and asking about their needs. Our results suggest that consideration of multiple perspectives, especially of hard to reach population groups, is important in order to identify and implement services to improve health in the area. Projections about the future demographic composition of the populations in an area should also be carefully considered when planning services, with special attention to the needs of those groups with the largest predicated population growth.

## Abbreviations

CE: Community Engagement
CM: Concept Mapping
LHIN: Local Health Integration Network
TC-LHIN: Toronto Central Local Health Integration Network
